# Impact of chronic alcohol consumption on inflammatory response and periapical bone resorption in induced apical periodontitis: a systematic review of animal studies

**DOI:** 10.1007/s10266-025-01122-4

**Published:** 2025-05-19

**Authors:** Isabel Silva Martins, Natália Pestana de Vasconcelos, Américo Santos Afonso, Ana Cristina Braga, Irene Pina-Vaz

**Affiliations:** 1 Faculty of Dental Medicine, University of Porto, 4200-393 Porto, Portugal; 2 Faculty of Medicine, CINTESIS, University of Porto, 4200-319 Porto, Portugal; 3 Health Science Faculty, University Fernando Pessoa, 4200-150 Porto, Portugal; 4 ALGORITMI Research Centre, LASI, University of Minho, 4710-057 Braga, Portugal

**Keywords:** Alcoholism, Animal model, Apical periodontitis, Endodontics, Pro-inflammatory cytokines

## Abstract

Alcohol consumption is linked to reduced bone mass and strength due to an imbalance in bone remodeling, characterized by decreased bone formation and an increased inflammatory response. Apical periodontitis affects the tissues surrounding the teeth and may also influence the systemic immune response by altering inflammatory marker levels. This systematic review examined how alcohol consumption affects the severity of experimental apical periodontitis in animal models. Following PRISMA guidelines, studies were sourced from three databases (PubMed, Scopus, and Web of Science) up to January 26, 2025. The included studies analyzed inflammation and/or bone resorption in animals with induced apical periodontitis, comparing alcohol-exposed groups to well-defined controls. Study quality was evaluated using the Animal Research: Reporting of In Vivo Experiments (ARRIVE) guidelines and the risk of bias was analyzed using the Systematic Review Centre for Laboratory Animal Experimentation (SYRCLE) tool. Of 135 screened records, 7 studies met the inclusion criteria. These studies were of moderate quality with a moderate risk of bias. Findings revealed that chronic alcohol consumption exacerbates inflammation and bone resorption in rats with experimental apical periodontitis, with effects increasing in a dose-dependent manner. The evidence suggests that chronic alcohol consumption amplifies the periapical inflammatory response in animal models, potentially accelerating apical periodontitis progression. These results highlight the need for further prospective studies to explore the broader implications of alcohol consumption in the context of apical periodontitis. This systematic review is registered on the International Prospective Register of Systematic Reviews (PROSPERO; CRD42024589226).

## Introduction

Alcohol use disorder poses a significant public health concern due to its far-reaching medical, psychological, and social consequences. It accounts for 5.3% of global deaths, and the World Health Organization has identified excessive alcohol consumption as a leading cause of preventable mortality [1, 2]. The health impacts of alcohol are shaped by both the total amount consumed and drinking patterns, affecting multiple systems in the body. Chronic alcohol consumption is strongly linked to cardiovascular and liver diseases, as well as cancers, including breast cancer, even at moderate intake. These harmful effects result from its toxicity to organs, potential to cause dependence, and psychoactive influence [2]. In addition to systemic impacts, alcohol has significant effects on oral health. Heavy episodic drinking impairs alveolar bone quality, contributing to or worsening periodontitis [3]. Chronic alcohol use further exacerbates periodontal disease by weakening the immune system, reducing resistance to pathogens, and promoting microbial imbalances in the oral cavity. The sugar content of alcoholic beverages and reduced salivary flow foster bacterial growth, increasing the risk of periodontal disease and dental caries [4].

Apical periodontitis (AP) is a prevalent inflammatory condition that impacts the periodontal ligament and adjacent tissues. It is frequently associated with untreated dental caries—its primary etiologic factor. AP is reported to affect a significant proportion of the adult population, with some studies estimating a prevalence of up to 52% [5]. It is characterized by alveolar bone resorption around the infected tooth and can influence systemic immune responses by altering levels of inflammatory markers such as C-reactive protein, tumor necrosis factor-alpha (TNF-α), IL-6, and IL-1 [6–8]. Systemic conditions also elevate these inflammatory mediators, contributing to a hyperinflammatory state that may impact AP’s progression and prognosis [9, 10]. Notably, chronic heavy alcohol consumption further exacerbates these inflammatory processes, intensifying nuclear factor kappa-B ligand (RANKL) activation, increasing tartrate-resistant acid phosphatase (TRAP)-positive cells, and raising TNF-α levels. These responses result in more severe alveolar bone loss, reduced pulpal blood flow, and greater periodontal damage, all dose dependent [11, 12].

Poor oral hygiene and neglect of dental care, common in individuals with high alcohol intake, compound the risk of dental caries and AP [13]. Despite the well-documented connection between alcohol and periodontal disease, the specific role of alcohol in AP development remains poorly understood. Animal studies have shown elevated inflammatory markers associated with chronic diseases in the presence of AP [7, 14, 15]. However, the association between AP and conditions such as cirrhosis or liver transplant candidates where chronic alcohol consumption could play a critical role is still unclear [10].

While clinical studies remain the gold standard for evaluating therapeutic interventions, animal studies are essential exploratory tools for understanding histological changes that cannot be routinely assessed in clinical settings. Based on current evidence, chronic alcohol consumption is hypothesized to influence AP progression by impairing osteoblast activity and intensifying inflammatory responses. This study systematically reviewed animal studies that examined the inflammatory reactions and periapical bone resorption associated with AP development, assessed at histological, radiographic, or serum levels in in vivo animal models.

## Materials and methods

### Protocol and registration

This systematic review followed the recommendations of the 2020 Preferred Reporting Items for Systematic Reviews and Meta-Analyses (PRISMA) guidelines [16]. The protocol herein presented was registered at PROSPERO International Prospective Register of Systematic Reviews (registration number: CRD42024589226).

### PICO question

The Population–Intervention–Comparator–Outcomes (PICO) strategy was defined as follows:

P (population): animals with experimental AP.

I (intervention/exposure): administration/consumption of alcoholic solution.

C (control/comparator): animals with experimental AP and non-alcohol consumption.

O (outcome): severity of AP (intensity of inflammatory infiltrate and bone resorption/bone density).

Two focused questions were addressed following the PICO format:

1. Does chronic alcohol consumption influence the inflammatory reaction associated with AP development in in vivo animal models?
2. Does chronic alcohol consumption influence the periapical bone resorption associated with AP development in in vivo animal models?

### Study selection criteria

*Inclusion criteria:* Animal studies; studies assessing the effects of chronic alcohol consumption vs. no alcohol consumption on the severity of induced AP; studies that include a control group with induced experimental AP that did not receive alcohol; articles written in English; studies using experimental models of AP and describing the characteristics of the animals used (animal type and species) and experimental methods used (type of disease induced, induction method, type and modality of its administration, outcome measurements and statistics); studies assessing outcomes of interest, namely the level of inflammatory reaction and/or bone resorption of experimental AP associated with alcohol consumption.

*Exclusion criteria:* Repeated findings; meta-analyses; scoping, systematic, or narrative reviews; meeting abstracts; case series; case reports; letters; editorials; studies that do not provide details on the materials and methodology as outlined in the inclusion criteria; control group not clearly defined or not meeting the study’s requirements; studies that include animals with comorbidities or under treatment.

### Search strategy

The search was carried out on January 26, 2025, on PubMed (Medline), Scopus, and Web of Science. The electronic search combined Medical Subject Heading (MeSH) terms and text words (tw). The Boolean operators “AND” and “OR” were used to create the search strategy (Table 1). No language or publication date restrictions were applied.

**Table 1:** Search strategy

Database	Search strategy	Findings
PubMed	#1 (apical periodontitis OR periapical condition OR periapical inflammation OR periapical lesions OR periapical pathology OR periradicular lesions)	12,945
	#2 (alcoholism OR alcohol OR alcohol consumption OR chronic alcohol consumption)	1,211,509
	#1 and #2	120
Scopus	#1 (alcoholism OR alcohol OR “alcohol consumption” OR "chronic alcohol consumption")	1,052,238
	#2 (“apical periodontitis” OR “periapical condition” OR “periapical inflammation” OR “periapical lesions” OR “periapical pathology” OR “periradicular lesions”)	7,151
	#1 and #2	32
Web of Science	#1 (alcoholism OR alcohol OR “alcohol consumption” OR “chronic alcohol consumption”)	780,693
	#2 (“apical periodontitis” OR “periapical condition” OR “periapical inflammation” OR “periapical lesions” OR “periapical pathology” OR “periradicular lesions”)	6,009
	#1 and #2	20

Two reviewers (I.M. and N.V.) independently evaluated the titles and abstracts of the search results and excluded papers that did not meet the eligibility criteria. When the title and abstract did not provide enough information to determine a study’s eligibility, the full text was reviewed. A third author, a senior researcher (I.P.V), resolved any disagreements and made the final decision regarding study inclusion.

### Data extraction

The following data were extracted and registered from each included study: name of the first author, year published, strain (species), sample, disease induction (time), administration, outcome measurements, and main results.

### Quality assessment

A quality assessment of animal studies was performed according to the updated Animal Research: Reporting of In Vivo Experiments quality guidelines [17]. It considered the “ARRIVE Essential 10” items, which are the minimum reporting requirements, and the “Recommended Set”. Two reviewers (I.P.V. and A.A.) scored each item independently, resolving any differences by discussion.

### Risk of bias

The risk of bias in the included studies was evaluated using the Systematic Review Centre for Laboratory Animal Experimentation (SYRCLE) tool, developed by the Cochrane Collaboration [18]. The risk of bias was judged as low, high, or unclear based on the answer to each of the ten entries of SYRCLE’s RoB tool: “yes” indicated a low risk of bias; “no” indicated a high risk of bias; “unclear” meant that insufficient details were reported concerning the item. Two reviewers (A.C.B. and A.A.) scored each item independently, resolving any differences by discussion.

### Data analysis

The clinical outcomes were summarized qualitatively, and a descriptive review was conducted.

## Results

### Literature search process and study selection

**Fig. 1:**
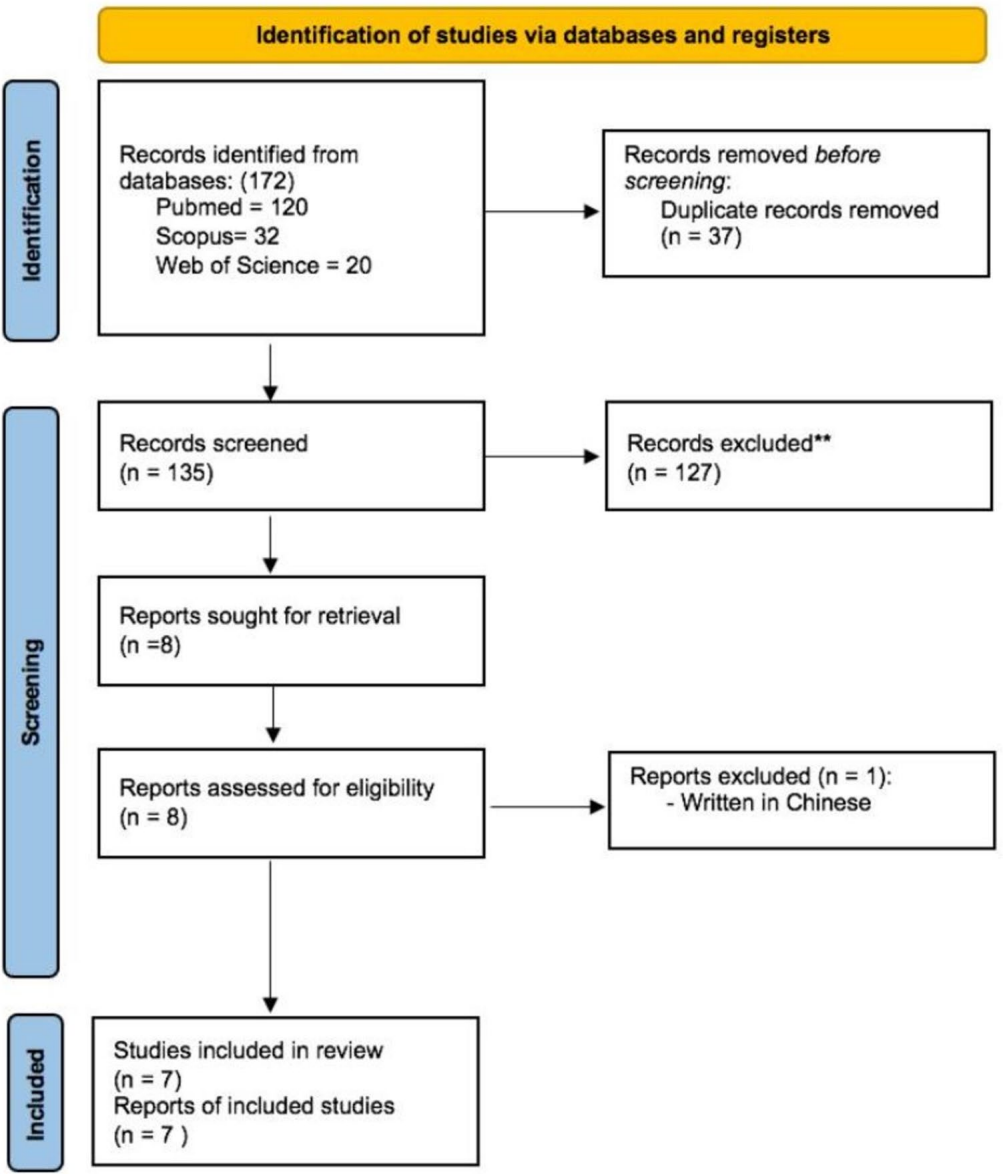
PRISMA (Preferred Reporting Items for Systematic Reviews and Meta-Analyses) flow diagram of the study search and identification of relevant studies

After searching the three databases, 172 articles (120 from PubMed, 32 from Scopus, and 20 from Web of Science) were identified, of which 37 duplicates were excluded. The remaining 135 relevant titles and abstracts were reviewed and screened based on the established selection criteria, leading to the exclusion of 127 articles. After a full-text review of the remaining eight articles, one more was excluded [19] (Fig. 1).

### Characteristics of the included studies

#### Animals included and groups of study

The seven selected reports were published between 2019 and 2024. All studies included male Wistar rats (Rattus norvegicus, Wistar strain) aged between 2 and 4 months, with initial weights between 200 and 340 g. There was always a control group of rats with induced AP without alcohol consumption and another group of rats to whom an alcoholic diet was introduced associated with experimental AP. Two studies [20, 21] also included groups with nicotine administration, and a third one [22] had other groups subjected to red wine or polyphenols. However, these additional groups did not prevent the data analysis of the outcomes of interest in the review. In all the studies, AP was induced through pulp exposure for 28 days in the first left lower molars in rats exposed to alcohol consumption (experimental groups). In one study [23], pulp exposure was also induced in the first lower right molar.

#### Alcohol administration/consumption

In the two studies by Pinto et al*.* [20, 21], animals were subjected to alcohol consumption by self-administration with weekly successive increments of 5% in the alcohol concentration until a final concentration of 25%. In two of the studies led by Dal-Fabbro et al*.* [24, 25], alcohol consumption began on day one with a 5% (v/v) alcohol/water solution as the sole source of hydration, which was gradually increased to 20%. In a third experiment led by Dal-Fabbro et al*.* [25], the animals received different alcohol concentrations per group (5%, 10%, 15%, and 20%, respectively) as their exclusive available liquid source. In the most recent study led by Dal-Fabbro et al*.* [22], the animals from the alcohol group received 4.28 mL/kg of body weight of an alcoholic solution containing 12.5% alcohol by volume. Finally, in Matos-Sousa study [23], a high intensity drinking ethanol protocol was established, consisting of 4 cycles with 3 days on and 4 days off per week of ethanol administration, at a concentration of 20% w/v, 3 g/kg/day via orogastric gavage.

Table 2 presents the main data extracted from the included studies.

**Table 2:** Characteristics of the included studies (*AST, aspartame aminotransferase; **ALT, alanine aminotransferase; IL, interleukin; Micro-CT, micro-computed tomography; AP, apical periodontitis; HIF, hypoxia-inducible factor)

Authors, year	Strain (species)	Sample	AP induction (timing and teeth)	Alcohol administration	Outcome measurements and methods	Main results
Pinto et al*.* 2024 [20]	Wistar rats (*Rattus norvegicus*, Wistar)	28 (n = 7)	Pulp exposure to the oral environment for 28 days (left^t^ 1st mandibular molars)	Self-administration of successive increases of 5% in the concentration weekly, until a final concentration of 25% alcohol	Levels of cytokines (Luminex assay), biochemical markers and metabolites (biochemical and metabolomic analysis of serum samples) Percent bone volume, bone mineral density, trabecular thickness, trabecular separation and trabecular number of the periapical bone (micro-CT)	Serum biochemical analysis: alcohol oral administration groups showed higher levels of pro-inflammatory cytokines: IL-1β, IL-6 and TNF-α; higher levels of biochemical markers AST*, ALT** and alkaline phosphatase; higher levels of uric acid and bilirubin; lower calcium levels Serum metabolomic analysis: metabolomic analysis showed significant differences for several parameters Alcohol oral administration groups showed higher levels of lysine, ethanol, lipid n(GH_2_) II and pyruvate Micro-tomographic analysis: an altered bone pattern indicating lower percent bone volume, lower bone mineral density, lower trabecular thickness, higher trabecular separation, and lower trabecular number was observed
Pinto et al*.* 2020 [21]	Wistar rats (*Rattus norvegicus*, Wistar)	28 (n = 7)	Pulp exposure to the oral environment for 28 days (left^t^ 1st mandibular molars)	Self-administration of successive increases of 5% in the concentration weekly, until a final concentration of 25% alcohol	Histopathological evaluation and immunohistochemistry for RANKL and PTHrP Area, volume, and major diameter of the periapical lesions (micro-CT)	Histopathological evaluation: alcohol oral administration presented a more intense inflammatory infiltrate the periapical region, and more extensive areas of bone resorption, compared to the control Immunohistochemistry: alcohol groups showed a more pronounced immunoreaction against RANKL and PTHrP antibodies Micro-tomographic analysis: periapical lesions with larger volume and area
Dal-Fabbro et al*.* 2019 [24]	Wistar rats (*Rattus norvegicus*, Wistar)	32 (n = 8)	Pulp exposure to the oral environment for 28 days (left 1st mandibular molars)	Self-administration of successive increases in the concentration, starting at 5% until it reached 20% of alcohol solution	Histological processing for histopathological and RANKL, OPG, TRAP, and HIF-1α	Alcohol oral administration increased the median score of inflammatory infiltrate The score values for RANKL, HIF-1a, and TRAP were higher Significantly more areas of bone resorption
Dal-Fabbro et al*.* 2019 [26]	Wistar rats (*Rattus norvegicus albinus*)	40 (n = 8)	Pulp exposure to the oral environment for 1 week (left^t^ 1st mandibular molars)	Self-administration of successive increases in the concentration an aqueous EtOH solution (5%, 10%, 15% e 20%) as their exclusive available liquid source	Histopathological analysis and immunohistochemistry: bone resorption areas and inflammatory reaction (inflammation and osteoclast markers)	Alcohol oral administration increased the chronic inflammatory infiltrate Alcohol oral administration at 15% and 20% concentrations increased the inflammatory infiltrate 15% and 20% alcohol concentrations and showed the highest immunolabeling pattern for RANKL and the lowest for and the lowest for OPG Increased AP size, with dose-dependent relationship
Dal-Fabbro et al*.* 2019 [25]	Wistar rats (*Rattus norvegicus albinus*)	32 (n = 8)	Pulp exposure to the oral environment for 1 week (left^t^ 1st mandibular molars)	Self-administration of successive increases in the concentration, from 5% alcohol solution until it reached 20%	Blood samples through a cardiac puncture Histopathological analysis—examination of the inflammatory infiltrate (scores) Radiographic density (digital radiographic images) in the periapical area	Calcium levels remained constant in all groups (*P* > 0.05). Alcohol + AP Alcohol oral administration increased phosphorous level Three animals in the experimental group exhibited a severe inflammatory reaction, whereas the animals in the control group (AP without alcohol) did not demonstrate any reaction (*P* < 0.05) Alcohol oral administration decreased the radiographic density
Dal-Fabbro et al*.*2021 [22]	Wistar rats (*Rattus norvegicus albinus*)	32 (n = 8)	Pulp exposure to the oral environment for 30 days (left^t^ 1st mandibular molars)	Self-administration of 4.28 mL/kg body weight of an alcoholic solution containing 12.5% alcohol	Median score of inflammatory infiltrate Micro-CT (resorption value)	Alcohol oral administration exacerbated the inflammatory process A lower median score of immunolabeling for **IL-1 beta** and a superior immunolabeling for **TRAP** was observed Alcohol oral administration did not influenced the mean value of lesion volume
Matos-Sousa et al*.* 2024 [23]	Wistar rats (*Rattus norvegicus)*	32 (n = 8)	Pulp exposure to the oral environment for 28 days (left and right 1s mandibular molars)	High-intensity ethanol drinking (3g/kg 20% w/v) by orogastric gavage for 3 consecutive days, followed by 4 days of withdrawal for 4 weeks	Histology and immunochemistry: changes in the inflammation profile of the disease: -histopathological analysis -pattern of bone loss -assessment of the collagen present Micro-CT analysis: -lesion volume -bone quality (bone volume and trabecular bone assessments)	Ethanol consumption heightened tissue deterioration Reduction of the collagen content of the alveolar bone Increased periapical lesion volume Reduced the percentage bone volume Modulation of the trabecular bone structure (reduction of the thickness of the trabeculae)

### Quality assessment

The ARRIVE quality assessment of the included studies provided the following findings about the selected studies:

- Concerning ARRIVE Essential 10 guidelines, all seven studies fulfilled the requirements, except for blinding. In three of the studies [23, 24, 26], outcome assessors for part of the investigation were reported to be blinded.

- Regarding the ARRIVE complementary Recommended Set, one study [21] did not mention the random distribution of animals, while the remaining six studies lacked details about randomization. Moreover, none of the studies mentioned animal care and monitoring or generalization/translation.

Most of the minimum required items and the vast majority of those from the Recommended Set were addressed in the selected articles. Therefore, these were considered high-quality studies.

### Risk of bias

Table 3 summarizes the SYRCLE risk of bias assessment. The seven studies selected were considered to have a moderate risk of bias.

**Table 3:**
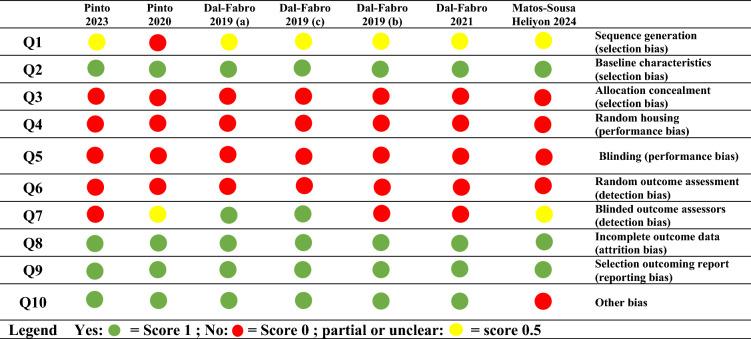
Risk of bias of the included studies (according to the Systematic Review Centre for Laboratory Animal Experimentation Risk of Bias tool)

### Answers to the review questions

Regarding the first review question about the inflammatory reaction impact, the alcohol-exposed groups of all studies exhibited higher inflammatory response scores. These groups showed increased levels of IL-1β, IL-6, and TNF-α [20], enhanced immunoreactivity to RANKL [21, 24], elevated HIF-1α [24], a higher number of TRAP-positive cells per mm, the strongest immunolabeling pattern for RANKL and the lowest immunolabeling for OPG [26], and/or a higher mean number of TRAP-positive cells [22], compared to the control group.

The second review question focused on periapical bone resorption associated with induced AP. The included studies consistently reported that alcohol exposure was associated with lower bone density and larger periapical lesions as assessed by micro-CT [20, 22] or digital radiographic images. Two studies led by Dal-Fabbro [24, 26] demonstrated increased bone resorption, reflected by higher RANKL and TRAP scores in the alcohol-exposed groups compared to controls. In addition, a synergistic effect between AP and alcohol intake was suggested by Matos-Sousa [23], which resulted in greater collagen degradation and impaired bone tissue repair mechanisms.

## Discussion

Chronic alcohol consumption significantly affects vital organs such as the liver and kidneys, leading to impaired function that disrupts bone metabolism [27]. AP is associated with elevated systemic inflammatory markers, resulting in the destruction of periradicular tissues, including pathological bone resorption.

This systematic review evaluated studies examining the inflammatory response and periapical bone resorption in in vivo animal models, simulating chronic alcohol consumption alongside AP development. Key findings from the seven included studies revealed that animals exposed to both chronic alcohol consumption and AP exhibited heightened levels of inflammatory markers and altered bone structure, characterized by increased resorption and reduced density, compared to controls (AP without alcohol). These effects were dose-dependent, with higher alcohol concentrations (15% or 20% v/v) producing more severe outcomes than lower concentrations (5% or 10% v/v) [26].

Animal studies on light alcohol consumption are scarce. While Yamamoto et al*.* [28] observed no detrimental effects on liver function with a 5% ethanol consumption, Maurel et al*.* [29] reported that prolonged moderate alcohol intake (8%–20%) adversely impacted bone remodeling in rats, in contrast to the negligible effects of short-term low-concentration exposure. Dal-Fabbro et al*.* [26] demonstrated a dose-dependent relationship, where 15% and 20% alcohol concentrations caused marked inflammatory reactions and bone resorption compared to lower doses.

In humans, chronic alcohol consumption is classified as light (1–10 g of ethanol/day), moderate (11–30 g/day), or heavy (> 30 g/day) [30]. Differentiating between these levels is crucial, as such light consumption may enhance bone density, while a heavy intake is linked to secondary osteoporosis and other organ damage [31]. In addition, a binge drinking pattern among adolescents and young adults is an emerging concern. Simulating that pattern in rats with 20% alcohol has been shown to cause damage to the alveolar bone, reducing the ratio of bone-to-tissue volume ratio in the alcohol-exposed group, with further intensification in the alcohol protocol associated with AP [23].

Variations in the effects of different alcoholic beverages on bone metabolism have also been highlighted, likely due to their unique constituents, such as polyphenols in wine or silicon in beer. In addition, factors like age and sex may further influence these effects [22, 32].

Findings from Pinto et al. [20] in which rats were exposed to successive increases in alcohol concentration up to 25%, corroborated earlier results [21]. These studies demonstrated that chronic alcohol consumption led to larger periapical lesions, greater inflammatory infiltrates, and systemic changes that exacerbated bone loss in AP models. The associated mechanisms involved altered cytokine levels, biochemical markers, and metabolites.

To model chronic alcohol intake in rats, alcohol concentrations of at least 20% were considered sufficient [26, 33]. Most studies included in this review employed spontaneous alcohol consumption, a wide accepted method for simulating addiction [34], with some protocols reaching concentrations of up to 25%.

Rats exposed to chronic alcohol consumption exhibited elevated biomarkers of liver damage, including aspartate aminotransferase (AST), alanine aminotransferase (ALT), and alkaline phosphatase [25], as well as increased bilirubin levels [20]. Elevated uric acid levels were also noted [20], likely due to alcohol’s purine content impairing renal excretion [35, 36]. Metabolomic analysis revealed reduced serum glycine and phosphocholine levels, further implicating systemic disruptions in bone formation pathways [37].

Bone homeostasis depends on a dynamic balance between osteoblasts, which form bone, and osteoclasts, which resorb it. Chronic inflammation promotes osteoclastogenesis via cytokines such as IL-1β, IL-6, IL-7, and TNF-α which disrupt bone remodeling by increasing RANKL expression and reducing OPG levels. RANKL binds to RANK on osteoclast precursors, stimulating their activation and promoting bone resorption. OPG, acting as a decoy receptor for RANKL, modulates this process, preserving bone integrity [38, 39]. Excessive alcohol intake exacerbates these pathways, as demonstrated by increased RANKL [21, 24] and TRAP expression, reduced OPG levels [26], and elevated inflammatory cytokines [20] in alcohol-consuming groups. These effects correlate with greater periapical bone loss and larger lesion volume, as observed in Dal-Fabbro’s studies [24, 26].

The studies herein included showed the association of alcohol groups to a lower bone density with larger volume and area of periapical lesions [21, 22], as assessed either by micro-CT [20–22] or digital radiographic images [25].

Animal studies offer advantages, including enhanced control over the study population and the ability to conduct histological analyses. Despite their limitations, most of the research involving laboratory animals has employed an alcohol-feeding protocol lasting at least 4 to 6 weeks, aiming to replicate the harmful effects associated with chronic alcohol consumption in humans. Thus, it is widely accepted that alcohol administration for a period of 1 month or longer can be classified as chronic exposure [40]. Moreover, in the selected studies, the lack of significant variation in rat strains, weights, or ages helps to minimize potential bias associated with these factors.

The impact of alcohol consumption on oral health has long been recognized, with particular emphasis on its role in the development of oral cancer. Maserejian et al. [41] demonstrated that alcohol intake, regardless of beverage type or drinking pattern, is associated with an increased risk of oral premalignant lesions, reinforcing the recommendation to reduce alcohol consumption for oral cancer prevention. Beyond oncological concerns, alcohol consumption has also been linked to oral trauma, halitosis, periodontal disease, and caries. Grocock [42] highlighted the importance of assessing alcohol use in dental settings, recommending that dental professionals incorporate alcohol risk assessment and reduction advice into routine care. The authors emphasized that taking a thorough alcohol history from all patients and using dental visits as opportunities to deliver preventive messages could contribute significantly to public health. Despite this, barriers to implementing such strategies in practice remain, and further efforts are needed to promote alcohol prevention advice, including enhanced education, training, and structural support.

In this context, our findings provide additional evidence of the clinical relevance of alcohol consumption in dentistry. Chronic alcohol exposure also appears to exacerbate the inflammatory response associated with AP, potentially resulting in more severe or persistent periapical lesions and decreasing the predictability of endodontic treatment outcomes. Clinically, this highlights the importance of considering alcohol intake during diagnosis and treatment planning, including a thorough medical and social history and clear communication with patients regarding prognosis and tailored management strategies.

Although the seven included studies presented a moderate risk of bias, an important limitation is that most of them were conducted by only two research groups. This may limit the generalizability of the findings. On the other hand, the use of samples collected from the same animals across studies may reduce variability related to animal, caging, and experimental conditions, thereby minimizing certain sources of bias. Another limitation is that it remains uncertain whether similar findings would be observed if the AP was previously established, or which pattern of ethanol exposure poses the greatest harm. In addition, questions persist regarding the impact of ethanol consumption, combined with individual characteristics, on the progression of periapical lesions.

sTo strengthen the evidence base and enhance the clinical relevance of future findings, we highlight the need for well-designed studies using standardized methodologies and conducted across multiple research centers. Such approaches would improve the transparency, reproducibility, and applicability of results to clinical practice. In this context, systematic reviews play a crucial role in summarizing scientific evidence, identifying the limitations and biases of included studies, and avoiding unnecessary duplication of research. They also provide a solid foundation for developing well-designed studies with standardized methodologies. To better understand the relationship between alcohol consumption and AP, further research is essential.

## Conclusion

Chronic alcohol consumption, particularly at higher doses, significantly impacts bone metabolism by promoting inflammation, increasing osteoclast activity, and impairing bone remodeling. In addition, the collagen content of the alveolar bone showed a reduction in the presence of AP, accentuated with alcohol consumption. These findings underscore the importance of understanding alcohol’s dose-dependent and systemic effects to better address its impact on bone health and related inflammatory conditions.

## Data Availability

Original data are available from the corresponding author upon request.
